# Bilateral High Intra‐Abdominal Testes Successfully Treated With Multistage Fowler–Stephens Orchiopexy to Preserve Testicular Function

**DOI:** 10.1002/iju5.70059

**Published:** 2025-06-03

**Authors:** Akari Hiraguri, Yuichi Sato, Junya Hata, Yusuke Kirihana, Akihisa Hasegawa, Satoru Meguro, Kanako Matsuoka, Seiji Hoshi, Souichiro Ogawa, Yoshiyuki Kojima

**Affiliations:** ^1^ Department of Urology Fukushima Medical University School of Medicine Fukushima Japan

**Keywords:** Fowler–Stephens orchiopexy, intra‐abdominal testis, magnetic resonance imaging, nonpalpable testes, testicular development

## Abstract

**Introduction:**

A case of bilateral high intra‐abdominal testes successfully treated with multistage Fowler–Stephens orchiopexy is reported.

**Case Presentation:**

A 6‐month‐old boy with bilateral nonpalpable testes was diagnosed with a left intra‐abdominal testis located immediately caudal to the spleen on magnetic resonance imaging and laparoscopy. At the age of 4 years, diffusion‐weighted magnetic resonance imaging detected a structure immediately caudal to the liver, which was suspected to be the right testis. With a diagnosis of bilateral intra‐abdominal testes, one‐stage Fowler–Stephens orchiopexy was performed on the right testis at 5 years of age. After confirming its development, two‐stage Fowler–Stephens orchiopexy was performed on the left testis at 10 years of age.

**Conclusion:**

After confirming the development of the right testis that underwent one‐stage Fowler–Stephens orchiopexy, two‐stage Fowler–Stephens orchiopexy was completed on the left testis, resulting in the successful preservation of both testes and normal sexual development.

**Summary**

- A case of bilateral high intra‐abdominal testes which were located caudal to the spleen and the liver.
- Multistage Fowler–Stephens orchidopexy was performed, resulting in successful preservation of both testes and normal sexual development.

**Abbreviations**
FSO: Fowler–Stephens orchiopexy
hCG: human chorionic gonadotropin
MRI: magnetic resonance imaging

## 1 Introduction

Approximately 20% of undescended testes are nonpalpable, including intra‐abdominal testis, intra‐canalicular testis, vanishing testis, and testicular agenesis [1]. Fowler–Stephens orchiopexy (FSO) is a well‐established treatment for intra‐abdominal testes. It involves ligating the testicular vessels to stimulate collateral circulation, maintaining blood supply to enable the descent of the testis into the scrotum. In the two‐stage FSO, scrotal placement of the testis is performed at a later time after ligating the testicular vessels. Generally, high intra‐abdominal testes are defined as those located more than 3 cm from the internal inguinal ring [2, 3]. It has been reported that, in cases of high intra‐abdominal testes, the higher the position of the testis, the greater the likelihood of postoperative atrophy following orchiopexy [4]. Furthermore, if the contralateral testis is normal, and the testicular vessels are too short to maintain adequate blood supply, orchiectomy may be considered for a high intra‐abdominal testis [5, 6]. However, there is no consensus on the treatment strategy for bilateral high intra‐abdominal testes, because bilateral orchiectomy or even bilateral orchiopexy, if followed by bilateral testicular atrophy due to impaired blood supply, results in male hormone deficiency, thus preventing virilization during puberty. A case of bilateral high intra‐abdominal testes successfully treated with multistage FSO, resulting in preservation of bilateral testes and normal sexual development, is reported.

## 2 Case Presentation

**FIGURE 1:**
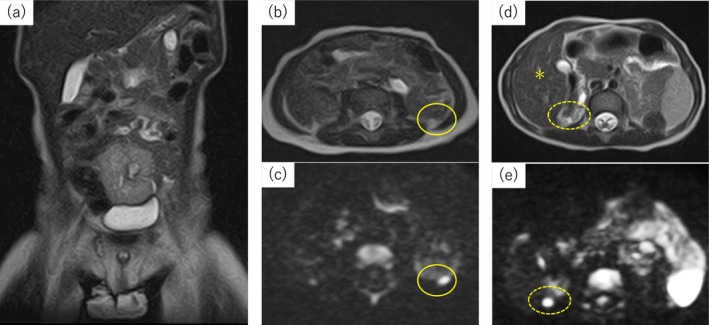
Magnetic resonance imaging (MRI) at 1 year and 2 months (a–c) and 4 years and 3 months of age (d, e). (a) T2‐weighted imaging, coronal section. There are no testes in the inguinal rings. (b) T2‐weighted imaging, axial section and (c) diffusion‐weighted imaging, axial section. The left testis is seen immediately caudal to the spleen (circle), but no right testis is seen. (d) T2‐weighted imaging and (e) diffusion‐weighted imaging show the structure (dotted circle) immediately caudal to the liver (asterisk), thought to be the right testis.

**FIGURE 2:**
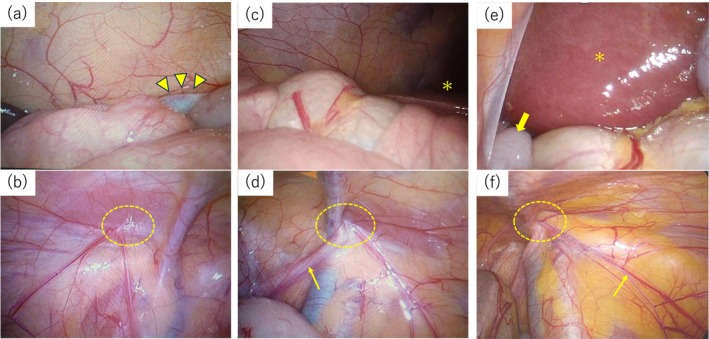
Laparoscopic images at 1 year and 5 months (a–d) and 5 years and 8 months of age (e, f). (a) Laparoscopy shows the left testis caudal to the spleen (arrow heads), (b) and does not show that the spermatic duct exits from the left internal inguinal ring (dotted circle). (c) It does not identify the right testis in the abdomen (asterisk: liver), (d) but shows that the spermatic duct (arrows) exits from the right internal inguinal ring (dotted circle). (e) The right testis (arrow) is seen immediately caudal to the liver (asterisk). (f) Collateral blood supply (arrow) runs from the right internal inguinal ring (dotted circle) to the right testis.

**FIGURE 3:**
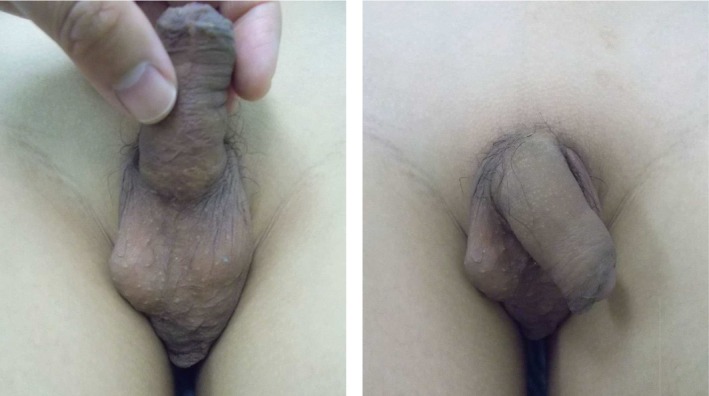
Appearance of the external genitalia at 12 years of age after bilateral orchiopexy. The patient has achieved well‐developed bilateral testes and normal sexual development with growth of the penis and pubic hair.

A 6‐month‐old boy with bilateral nonpalpable testes was referred. Chromosome analysis showed a normal karyotype, 46, XY. A human chorionic gonadotropin (hCG) stimulation test was positive. Magnetic resonance imaging (MRI) at 1 year and 2 months of age identified a structure with a diffusion‐weighted signal immediately caudal to the spleen, which was suspected to be a left testis, but no right testis was seen (Figure 1a–c). It also showed a right hypoplastic kidney and a left pelvic kidney. Laparoscopy was performed at 1 year and 5 months of age and identified the left testis in the same location as indicated by MRI (Figure 2a,b). However, the right testis was not identified, and the spermatic duct exited from the right internal inguinal ring (Figure 2c,d). As only a single high intra‐abdominal testis was identified, follow‐up was performed without orchiopexy, but with annual MRI, with priority given to achieving normal sexual development and masculinization in the future, while also explaining the risks of malignant transformation and future infertility to the patient's guardians. At 4 years and 3 months of age, diffusion‐weighted MRI showed a structure immediately caudal to the liver, which was undetected previously, and was suspected to be the right testis (Figure 1d,e). To confirm the presence of the right testis, laparoscopy was performed at 5 years and 8 months of age. At that time, the patient was positioned in the semi‐lateral position, unlike the supine position used in the first laparoscopy. Three trocars were placed to mobilize the intestinal tract, unlike the single trocar placed previously. After incising the retroperitoneum and mobilizing the ascending colon, the right testis was successfully identified immediately caudal to the liver with collateral blood supply running from the right internal inguinal ring to the testis (Figure 2e,f). Using this collateral blood supply, one‐stage FSO was performed on the right testis. After confirming the development of the right testis and increased serum testosterone, orchiopexy was performed on the left testis at 10 years of age. The left testis was identified caudal to the spleen. Due to insufficient collateral blood supply on the left side, two‐stage FSO was performed. After bilateral orchiopexy, the patient showed well‐developed bilateral testes and successful onset of sexual development with growth of the penis and pubic hair (Figure 3).

## 3 Discussion

A case of bilateral high intra‐abdominal testes located immediately caudal to the liver and spleen was presented. To the best of our knowledge, there have been no previous reports of successful preservation of both testes after bilateral orchiopexy for bilateral high intra‐abdominal testes.

Generally, the high position of the testes reduces the success rate of orchiopexy and increases the risk of postoperative atrophy [4]. Therefore, the highest priority was placed on achieving sexual development without testicular atrophy by multistage FSO. However, delaying orchiopexy for intra‐abdominal testes and opting for observation poses risks, such as malignancy or impaired spermatogenesis. However, only a few cases of malignant transformation of intra‐abdominal testes in children under 10 years of age were previously reported [7]. In addition, since it was reported that ligation of the spermatic vessels during orchiopexy for intra‐abdominal testes is associated with a significant reduction of spermatogonia [8], improvement of spermatogenesis following FSO might be limited. Based on these reports, it was considered that the risk of malignancy or acquisition of spermatogenesis following FSO was low; thus, secondary sexual development was prioritized, and multistage FSO was performed.

**FIGURE 4:**
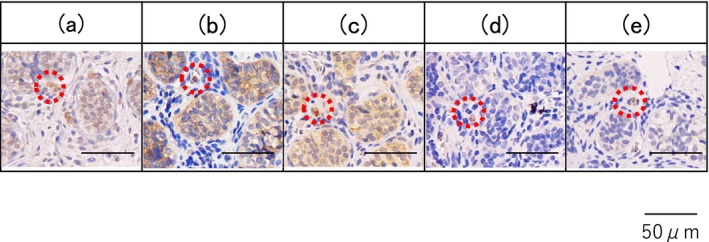
Flow of treatment for this case. First, the patient was diagnosed as monorchism by MRI and laparoscopy, so he was followed‐up without orchiopexy. But following MRI showed a left testis, so it was diagnosed as bilateral high intra‐abdominal testes. Multistage FSO was performed.

We first performed FSO on the right testis because the vessel used as collateral blood supply was stronger than the left side. After confirming the development of the right testis and increased serum testosterone, FSO was performed on the left testis. The patient successfully achieved well‐developed bilateral testes and normal sexual development by multistage FSO (Figure 4).

Laparoscopy is typically the diagnostic tool for nonpalpable testes, whereas MRI alone is generally insufficient to rule out the need for surgery [9]. In the present case, however, the first laparoscopy did not identify the right testis, but subsequent MRI identified it caudal to the liver. It might be difficult to identify intra‐abdominal testes by laparoscopy, particularly if the testis is located high in the abdominal cavity, or if the vas deferens exits from the internal inguinal ring, as in the present case. MRI could be a valuable adjunct tool for preoperative diagnosis of high intra‐abdominal testes.

## 4 Conclusions

A case of bilateral high intra‐abdominal testes was described. Multistage FSO successfully preserved both testes and achieved normal sexual development. MRI proved to be a useful adjunct tool for preoperative diagnosis of intra‐abdominal testes.

## Disclosure

The authors have nothing to report.

## Ethics Statement

The authors have nothing to report.

## Consent

The authors have nothing to report.

## Conflicts of Interest

The authors declare no conflicts of interest.
