# Correction to “Bilateral High Intra‐Abdominal Testes Successfully Treated With Multistage Fowler–Stephens Orchiopexy to Preserve Testicular Function”

**DOI:** 10.1002/iju5.70087

**Published:** 2025-09-03

A. Hiraguri, Y. Sato, J. Hata, et al., “Bilateral High Intra‐Abdominal Testes Successfully Treated With Multistage Fowler–Stephens Orchiopexy to Preserve Testicular Function,” *IJU Case Reports* 8, no. 4 (2025): 427–430.

The image originally published as Figure 4 contains an error; the correct version of Figure 4 is as follows.

**FIGURE 4:**
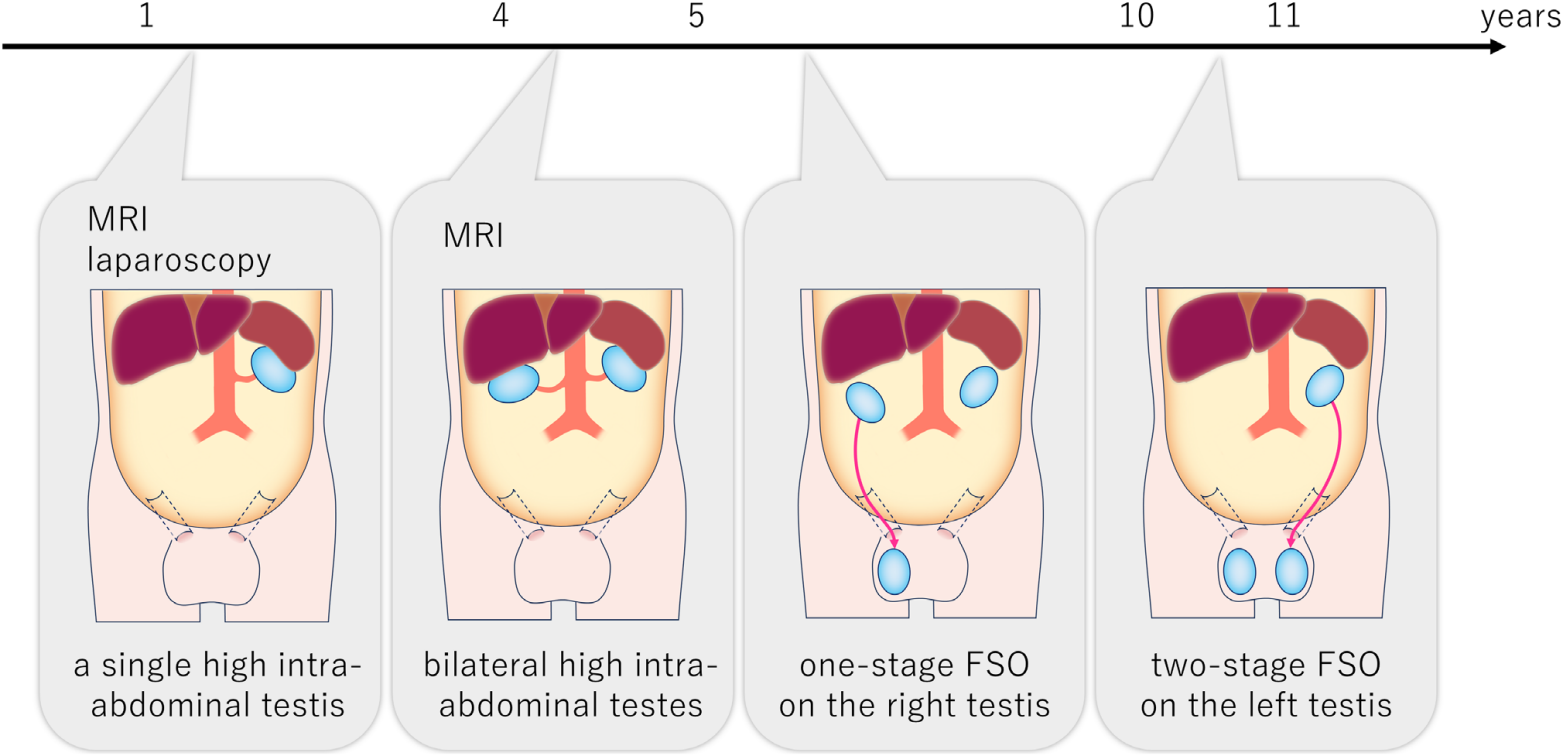
Flow of treatment for this case. First, the patient was diagnosed as monorchism by MRI and laparoscopy, so he was followed up without orchiopexy. But following MRI showed a left testis, so it was diagnosed as bilateral high intra‐abdominal testes. Multistage FSO was performed.

We apologize for this error.

